# What are the effects of exergames on the mood states of older people? A systematic review of experimental studies, impacts on mental health and recommendations

**DOI:** 10.18632/aging.206361

**Published:** 2026-03-18

**Authors:** Camile de Bem Gaspar, Whyllerton Mayron da Cruz, Alexandro Andrade

**Affiliations:** 1 Laboratory of Sport and Exercise Psychology, Human Movement Sciences Graduate Program, College of Health and Sport Science of the Santa Catarina State University (UDESC), Florianópolis, Brazil

**Keywords:** electronic games, older adults, BRUMS, mental health, physical activity

## Abstract

Aging results from a process of loss of physical capacity and cognitive and emotional declines. The practice of physical exercises has been shown to be a factor in slowing down these declines, and maintaining physical and mental health. Among the exercise modalities that have been shown to be effective, exergames demonstrate positive potential for general health and, especially, for mental health, including mood. This study reviewed exergames’ effects on older adults’ mood states, following Systematic Reviews and Meta-Analyses protocols and with register Prospective Register of Systematic Reviews (CRD42024526448). This study aimed to analyze the effects of exergames on the mood states of older individuals. A literature search across PubMed, Embase, Web of Science, and Scopus identified 651 studies, with nine meeting the inclusion criteria, encompassing 325 participants aged 61 to 78.9 years. The results indicated that exergames positively impacted mood in older adults, reducing tension, anger, fatigue, confusion, and depressive symptoms, while promoting engagement, immersion, and socialization. No studies reported worsening mood, supporting exergames as a safe activity for this group. However, more long-term studies are suggested to strengthen the evidence.

## INTRODUCTION

People around the world are living longer, so the number and proportion of people aged 60 and over in the population is growing, and this number is predicted to increase to 1.4 billion by 2030 [1, 2]. Aging is a complex phenomenon, marked by a gradual and continuous process, characterized by its wide range of dimensions and the complexity of its transformations that vary from individual to individual [3–5]. An aspect frequently experienced in old age is the gradual decrease in physical and mental capacity, which promotes a reduction in social interactions in this population, and which has the potential to significantly affect the mental health, well-being, and mood of these individuals [6–10].

Physical exercise (PE) has been investigated as an alternative to treat, prevent, and delay aging, and it has been proven that PE acts as a protective factor for chronic non-communicable diseases and in the treatment of other common diseases in aging [10–15]. In addition, several studies have demonstrated the positive effects of different types of PE on mental health, anxiety, and depression in older adults [16–20].

Furthermore, regular physical activity brings substantial benefits to older adults, acting as a protective factor against the development of mental health problems and promoting improvements in mood. Studies show that a greater investment of time in physical activities is associated with even more significant benefits [6, 21, 22].

Exergames are a new way of practicing physical exercise, leading to high adherence, and presenting considerable physiological and mental results [23–25]. Recent studies indicate that exergames are emerging as a versatile tool for interventions in various domains, due to their accessibility, low cost, and significant results [26, 27]. Other research indicates that practicing exergames can improve the mood of children and adolescents, significantly increasing vigor when practiced in a group [28–30]. In adults, an increase in happiness was observed in students and university staff when practicing individually [31], in addition to providing a lower perception of fatigue during practice among young adults when compared to traditional aerobic dance [32].

These effects may occur as a result of the mechanisms brought about by the practice of exergames, which can consequently influence mood, through engagement, social interaction when the practice is collective, and cognitive stimulation itself. Studies indicate that engagement increases experience and pleasure in the game, improves mood, and is related to a greater likelihood of wanting to practice again at another time [33–35]. In addition, social interaction is another important component of exergames, especially for older people, since the practice of exergames has been shown to improve social well-being, decrease loneliness, and increase social connection between participants, which consequently influences the improvement in mood [36]. Finally, we can consider the cognitive stimulation that occurs from exergames that combine motor and cognitive activities, which can improve cognitive functions such as memory and processing speed, also alleviating depressive symptoms [37, 38].

Exergames represent an innovative and promising alternative to traditional physical activities for older adults, offering distinct benefits in terms of adherence, accessibility, and motivation. Studies indicate that the interactive and playful nature of exergames can significantly enhance adherence to physical exercise, making it more engaging and enjoyable for this population [33, 36, 37, 39]. Additionally, the accessibility of exergames is a key factor, as they can be performed at home, eliminating barriers such as transportation and mobility limitations, which was shown to be particularly relevant during periods of social restrictions, such as the pandemic [33–35]. Thus, exergames emerge as a valuable strategy for promoting physical activity and well-being, integrating physical, cognitive, and social benefits in an accessible and effective manner.

Furthermore, recent studies indicate that participation in exergames can have positive effects in older adults, with regular practice of these games contributing to improvements in mood, increased feelings of well-being, and promoting vitality in older adults, providing a fun and stimulating way to stay active [40–42]. In this context, with the exponential growth in scientific production in various methodological approaches and the increase in clinical trials investigating the effects of exergames in older adults [26, 43–48], it becomes essential to synthesize and critically evaluate this literature, to clarify the effects of exergames on mood states and determine the best way to incorporate them into physical exercise practice for older adults. This synthesis will provide new insights for physical education and health professionals, allowing for an evidence-based approach.

With a high degree of scientific evidence, systematic reviews and meta-analyses are considered the best sources for decision-making in clinical/professional practices, and are increasingly used and cited by researchers due to the growing volume of scientific literature [49]. Thus, the aim of the current study is to analyze, through a systematic review, the impacts of exergames on the mood states of older adults.

## RESULTS

**Figure 1:**
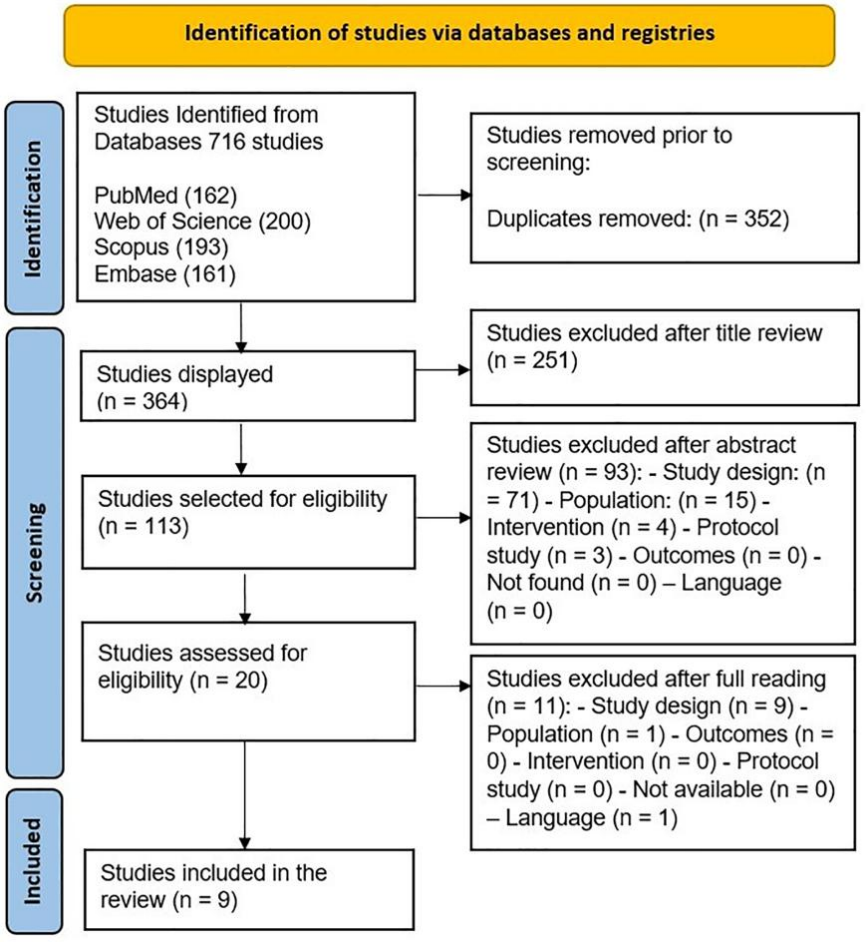
PRISMA flowchart illustrating the literature search and selection process.

In the first stage of the database search, 651 studies were identified, of which 331 duplicates were excluded. After reading the title and abstract, 20 articles remained and were selected for full reading. At the end of the selection process, nine studies met the eligibility criteria and were selected for synthesis (Figure 1 PRISMA flowchart).

### Characteristics of included studies

Among the studies included in the review, the oldest was published in 2013 [50] and the most recent were published in 2023 [40, 41, 51] (Table 1). The studies were carried out in Brazil [40, 42, 52, 53]; Italy [45]; the United Kingdom [50], Turkey [54]; and the United States [41, 51].

**Table 1:** Search strategy adopted for systematic review.

**Search terms**	**Descriptors**
Elderly	Elderly OR aged OR aging OR “aged, 80 and over” OR “older adults” OR “older women” OR “older men” OR senescence OR “oldest old” OR “old adults” OR nonagenarian^*^ OR octogenarian^*^ OR centenarian^*^ OR elder^*^ OR geriatr^*^ OR “older people” OR "older person" OR senior
Mood	Mood OR moods OR “mood states” OR brums OR “brunel mood scale” OR poms OR “profile of mood states” OR depress^*^ OR tense^*^ OR tension OR irrita^*^ OR annoyed OR hostil^*^ OR hate^*^ OR frustrate^*^ OR rage OR moody OR anxious^*^ OR temper OR vigor OR vigorous OR emotion^*^ OR anxiety OR distress^*^ OR anger^*^ OR angry OR happy^*^ OR sad OR sadness OR confus^*^ OR upset
Exergames	Exergames OR exergaming OR exergame OR exergames OR “gaming, active-video” OR “virtual reality exercise”

### Participants and instruments

The 9 included studies examined psychological aspects in 325 older adults, with sample sizes ranging from 14 [41] to 83 participants [53] (Table 1). Two studies analyzed only older women [40, 52]. Ages ranged from 61 ± 6 years to 78.9 ± 8.7 years. Several instruments were adopted, among which the Profile of Mood State (POMS) was used in two studies [40, 52].

### Interventions

Regarding the characteristics of the interventions, we observed several differences between the studies, making it impossible to develop a meta-analysis (Table 2). Two studies included Focus Groups, where the results were collected through interviews and group conversations [34, 35]. Regarding the comparisons performed in the selected studies, the categories identified were in relation to the level of physical activity of the individuals [44], to the control group, which practiced manual activity workshops [33], or that carried out goal setting and education on physical activity [44]. Three studies compared exergames with control groups [38, 45, 47] and two studies carried out only one intervention and compared the pre-intervention moment with the post-intervention moment [41, 44].

**Table 2:** Criteria for inclusion and exclusion.

		**Inclusion criteria**	**Exclusion criteria**
**P**	Population	Elderly ≥60 years	Studies including individuals younger than 60 years old
**I**	Intervention	Interventions using exergame	Studies not involving exergames or using non-interactive physical activities
**C**	Comparator	Among the exergame modalities, control group without exercise, with the same intervention, but different intensity, healthy individuals, other disease or disorder or not, individuals of different sexes	Studies without a comparator group or using an unrelated intervention
**O**	Outcomes	Effects of exergames on mood	Studies not assessing mood-related outcomes
**S**	Study design	Experimental, randomized, non-randomized, pre and post studies	Letters, editorials, commentaries, abstracts, conference proceedings, study protocols, case studies, review articles, non-experimental studies, cross-sectional or longitudinal studies

The most commonly used consoles were the Xbox 360 with Kinect [40, 42, 45] and the Nintendo Wii [50, 52, 54], and the most widely used exergame was Wii Fit [50, 52, 54], however a wide variety of exergames were included.

### Study quality

After assessing the quality of the studies, the studies obtained general and specific classifications of the study quality assessment criteria [55] (Table 3). The included studies obtained an average score of 8.11 (±1.05), ranging from 6 to 10. Based on this assessment, the quality of all studies was considered regular.

**Table 3:** Characteristics of participants and measures used to assess mood in the studies included in the review.

**References**	**Sample size and gender/by group**	**Age range/by group**	**Measurement (Instrument used)**
[51]	S(n) = 19 SL(n) = 1 EG(n) = 10 (M = 1; F = 9) CG(n) = 9 (M = 6; F = 3)	EG = 76,4 ± 7,6 years CG = 71,4 ± 3,9 years	Patient Health Questionnaire-8 (PHQ-8)
[40]	S(n) = 22 (F) SL(n) = 9 EG(n) = 9 CG(n) = 13	EG = 66,2 ± 4,6 years CG = 73,6 ± 8,0 years	Profile of mood state (POMS)
[41]	GF(n) = 14 SL(n) = 0 (M = 4; F = 10)	GF = 78,9 ± 8,7 years	Focus group interviews with 4–5 participants (Script included questions based on Diener et al.’s 2018 theory of subjective well-being)
[45]	S(n) = 57 SL(n) = 5 EG(n) = 38 (M = 9; F = 29) CG(n) = 19 (M = 6; F =13)	EG = 70,13 ± 3,73 years CG = 71,11 ± 3,72 years	Beck Depression Inventory II
[54]	S(n) = 44 SL(n) = 14 EG exergame(n) = 16 (M = 7; F = 9) EG physical activity(n) = 14 (M = 6; F = 8) CG(n) = 14 (M = 9; F = 5)	EG exergame = 72,25 ± 5,95 years EG atividade física = 75,14 ± 5,50 years CG = 73,86 ± 4,63 years	Hamilton Rating Scale for Depression (HRSD)
[53]	S(n) = 83 SL(n) = 0 AG(n) = 41 (M = 5; F = 36) SG(n) = 42 (M = 26; F = 16)	AG = 69,37 ± 5,56 years SG = 74,17 ± 8,6 years	Brunel Mood Scale (BRUMS)
[42]	S(n) = 36 SL(n) = 14 EG exergame(n) = 14 (M = 5; F = 9) CG aerobic exercise(n) = 22 (M = NI; F = NI)	EG = 61,7 ± 5,7^*^ years CG = NI	Focus group interviews
[52]	S(n) = 30 (F with chronic low back pain) SL(n) = 4 CG physical activity(n) = 14 EG exergame(n) = 16	S = 68 ± 4 years	Profile of Mood States (POMS)
[50]	S(n) = 20 SL(n) = 0 (M = 6 F = 14)	S = 61 ± 6 years^**^	Positive and Negative Affect Schedule (PANAS)

Abbreviations: n: number; S: samples; SL: sample loss; EG: experimental group; CG: control group; AG: active group; SG: sedentary group; M: masculine; F: female, NI: Not informed. ^*^This study enrolled 6 individuals aged 55 to 59 years. ^**^This study included individuals in its sample who were between 55 and 59 years old.

Most of the included studies presented common limitations, such as blinding of evaluators and the design of interrupted time series. This included the use of individual-level data to determine group-level effects, especially when the intervention was related to a specific group. Interobserver agreement for all items was 95.8%.

### Effects of exergames on the mood of older people

**Figure 2:**
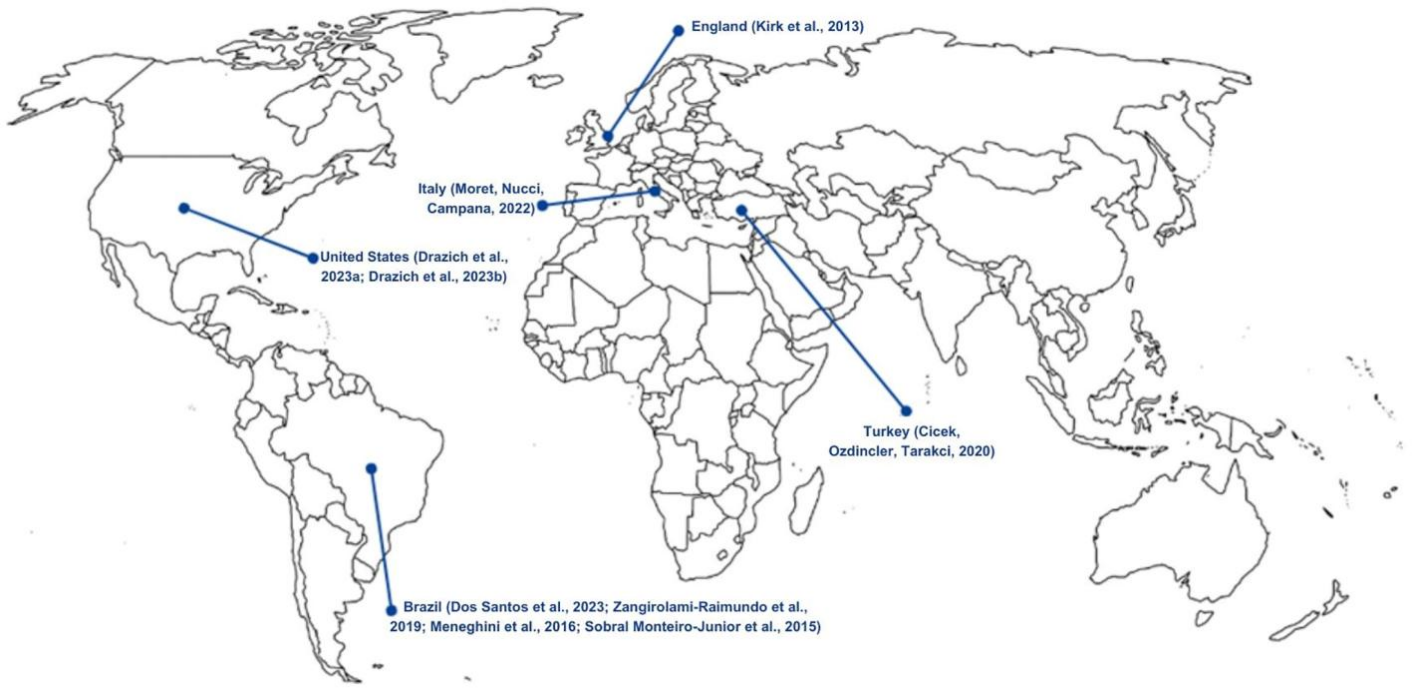
**Global geographic distribution of studies on the effects of exergames on mood states in older adults.** Country (Article references).

It was found that the effects of exergames on mood varied among positive, negative, or null, depending on the characteristics of the studies analyzed and the profiles of the participants. Six studies showed that the practice of exergames by older adults produced positive effects on mood [40, 42, 50, 51, 53, 54] (Figure 2), in three studies, no significant effects were observed [41, 45, 52], and positive effects in only one aspect of mood were observed in the study by [40]. The practice of exergames did not present a negative effect in any of the studies analyzed (Frame 1).

**Frame 1:** Effects of exergames on the mood of older people.

**References**	**Positive effects**	**It had no effect**	**Negative effects**
[51]	−	There was a positive, but not significant, influence of the program in reducing depressive symptoms in both groups.	−
[40]	Tension, depression, anger, fatigue and confusion	Vigor	−
[41]	Improved mood (game activity, feelings of immersion and socialization)	−	−
[45]	−	There was no statistical evidence of a beneficial effect of exergames on mood	−
[54]	Decrease in depression score in both experimental groups	−	−
[53]	Depressive symptoms influence exergame performance	−	−
[42]	There was a positive influence of the program on mood and emotions	−	−
[52]	−	There was no statistical evidence of a beneficial effect of exergames on mood	−
[50]	A single session of Wii activity can result in positive mood changes (mood improved before and after the session)	−	−

Regarding the results related to sex, there was no distinction of results by this variable in any of the studies analyzed that evaluated both sexes.

When analyzing mood variables, it is possible to observe that only two studies analyzed the variables tension, anger, fatigue, mental confusion, depressive symptoms, and vigor [40, 52], four studies analyzed only depressive mood symptoms [45, 51, 53, 54], three studies analyzed mood more comprehensively, related to positive and negative effects [50], and one in a qualitative way [42, 51], described as general mood (Frame 2).

**Frame 2:** Effects of exergames on mood variables in elderly people.

**References**	**Mood variables**						
	**Tension**	**Anger**	**Fatigue**	**Mental confusion**	**Depression**	**Vigor**	**Overall mood**
[51]					−		
[40]	↓	↓	↓	↓	↓	−	
[41]							↑
[45]					−		
[54]					↓		
[53]					−		
[42]							↑
[52]	−	−	−	−	−	−	
[50]							↑

Abbreviations: (↑): increased; (↓): decreased; (−): no significance.

Only two studies incorporated focus groups into their methodology (Frame 3). The interviews conducted to collect data brought a variety of enriching concepts to the research, since they were self-reported by the participants. In addition to presenting an improvement in mood, the interviews also highlighted other aspects, such as socialization, well-being, and the duration of the effects of exergames on mood.

**Frame 3:** Studies that used focus groups in their outcome assessments.

**References**	**Intervention**	**Results**
[41]	The exercise practiced was “I Am Dolphin”, which lasted 15 sessions of 60 minutes.	Playing exergames improved the mood of the older adults through the game activity, but after the activity ended, the participants reported that this improvement was short-lived. The feelings of immersion gave the individuals a perception that time did not pass during the practice and did not cause boredom during the activity. The socialization that the game brought went beyond the practice, as the older adults had the opportunity to comment on the game with their friends and family.
[42]	The practice was carried out in pairs and was made up of 36 sessions of 50 minutes. The games played included: athletics, bowling, boxing, skiing, football, tennis, table tennis, and some mini-games.	Playing exergames was compared by the older adults to “emotional therapy”, in addition to providing well-being to individuals. Thus, it was possible to observe that the practice of exergames positively influenced the mood and emotions of the older adults.

Although the practice of exergames produced effects on several mood variables, some studies focused specifically on the effects of exergames on depressive symptoms in older adults (Frame 4). Of these, only one study presented results that exergames provide a significant positive effect on the depression score [54], while the other two studies did not find significance in their results, but confirmed a positive trend.

**Frame 4:** Effects of exergames on depressive mood symptoms in older individuals.

**References**	**Intervention**	**Results**
[51]	The EG practice was carried out on a recumbent bicycle with virtual reality glasses. The CG received classes on the practice and importance of physical activity. The intervention included 16 sessions of 40 minutes.	On average, participants reported mild depressive symptoms. After the intervention, both groups presented reductions in their depressive symptoms by an average of one point, but this result was not significant. The program therefore had a positive influence on reducing depressive symptoms in both groups.
[45]	The EG only performed the exergame practice (Dr Kawashima: brain and body exercises) while the CG maintained their daily routine, the duration of the intervention was 8 sessions of 45 minutes.	A positive trend was observed in the measurement of depressive symptoms, showing that the practice of exergames reduces depressive symptoms, but there was no statistical proof of this evidence.
[54]	There were three groups in this intervention: the exergame group (Wii Fit Plus), the physical activity group (stationary bike cycling and treadmill walking), and the CG, who maintained their daily routine. The intervention included 16 30-minute sessions.	There was a significant positive effect on depression scores in both active groups, but the effects of Nintendo Wii Fit video games were more significant than conventional physical activity.

## DISCUSSION

**Figure 3:**
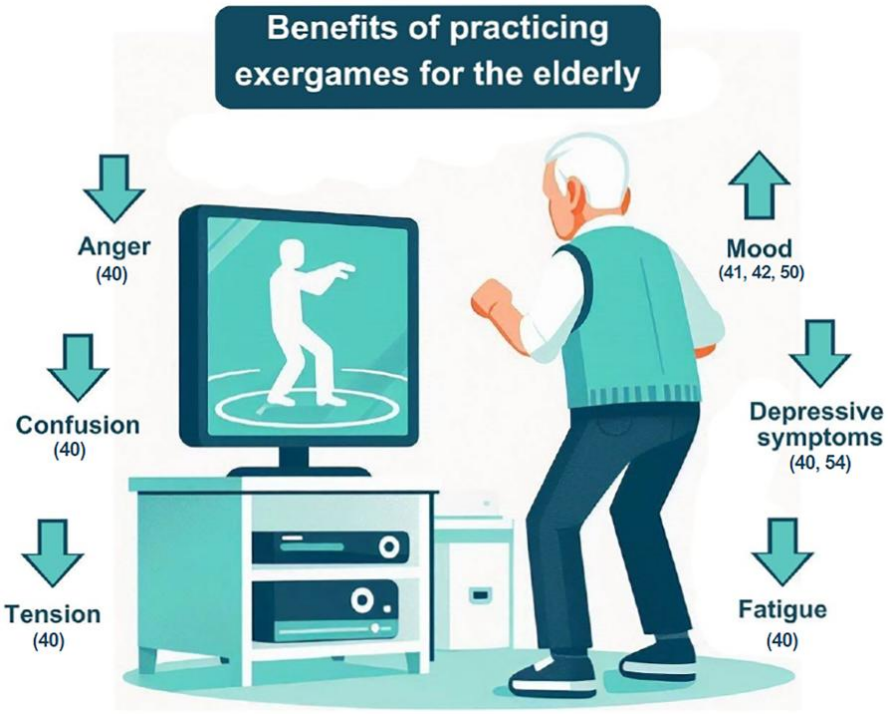
Benefits of practicing exergames on mood variables in the elderly.

The aim of the current study was to analyze the effects of exergames on the mood states of older individuals, by analyzing mood variables related to mental health and recommendations. In total, nine studies met the inclusion criteria. The main results showed that the practice of exergames provided positive effects on mood. Recent studies evaluated other psychological aspects, such as reductions in depression, anxiety, and apathy [56], which also presented positive results for the mental health of older individuals and which corroborate the results of the studies reviewed in the current work [57, 58]. These findings are summarized in Figure 3.

### Effects of exergames on the mood profile of older people and related variables

Our systematic review demonstrates, based on the analysis of the selected studies, that the practice of exergames produced positive effects on the mood of older adults, in addition to reducing depressive symptoms. None of the included studies demonstrated that the practice of exergames provided negative effects on the mood and mental health of older adults. These findings indicate that the practice of exergames can lead to a reduction in depression levels and improved quality of life [59], in addition to allowing for improved well-being and psychological, emotional, and social improvements [60]. It was found that the practice of exergames had positive effects on reducing anger, depressive symptoms, fatigue, tension, and mental confusion [40], however it did not have a positive effect on the vigor variable, which was not expected when we consider the existing evidence demonstrating positive impacts of exergames on the vigor of adults and children [61, 62]. However, in the study by [40], vigor was already high before the intervention.

Given the relevance of studies that analyze mood through six specific variables; depressive symptoms, mental confusion, tension, anger, fatigue, and vigor, it is crucial to reflect on these findings individually, however, only one of the nine studies included in this review presented the results separately by variables [40].

Depressive symptoms showed a significant reduction in the study that evaluated this variable through the Brunel Mood Scale (BRUMS) and presented the results of each mood variable individually [40]. Regarding the studies that evaluated this variable using other methods, one study observed a significant reduction [54], while the other four studies did not present significant results [45, 51–53]. These results are similar to those found in the literature when evaluating other populations [61, 63].

Significant reductions were generally observed in tension, anger, mental confusion, and fatigue [40], and these results are confirmed by the literature [29, 61, 63], however, one study showed an increase in fatigue after the practice of exergames by children [29], while another showed an increase in anger in the female population of the study [61], therefore, it is important to take into account that different populations and different games applied can influence the results. No significant results related to vigor were found [40]. These results diverge from those found in the literature, which show an increase in vigor after practicing exergames [28, 31].

The results of the current review point to the need for further studies that investigate the effects of exergames on the mood of older people, with the aim of establishing recommendations that include detailed guidelines and suggestions for maintaining a positive mood during and after practicing these games.

### Potential mechanisms underlying the effects of exergames on mood

The social interaction facilitated by exergames can help improve mood. Studies show that social engagement can reduce loneliness and increase social connection among participants, especially in older populations [64]. The social interaction fostered by these games can create a supportive environment that is beneficial for mood and overall mental health [57, 64]. Furthermore, practicing exergames in a group can foster positive attitudes towards others, contributing to a richer and more satisfying emotional experience [64].

Cognitive engagement is another potential mechanism that may explain the positive effects of exergames on mood. Playing exergames requires attention, decision-making, and problem-solving, which can stimulate cognitive activity and, consequently, improve mood [38, 65]. Studies show that engaging in cognitively challenging activities, such as exergames, can lead to greater feelings of vigor and happiness [29, 66]. Additionally, cognitive engagement can increase immersion and enjoyment during gameplay, which is associated with more positive mood experiences [30, 67].

Thus, both social interaction and cognitive engagement are potential mechanisms that could explain the positive effects of exergames on mood. Social interaction can improve emotional well-being by fostering social connections and support, while cognitive engagement may increase satisfaction and vigor through mental challenges. Exploring these mechanisms in greater depth can enrich the discussion on the benefits of exergames for mental health.

### Sex differences in experimental studies with exergames in older individuals

Regarding the characteristics of the participants, the majority (6 out of 9 studies) conducted the experiments with the participation of female individuals [41, 45, 50, 51, 53], including two studies that only selected female participants [40, 52].

Although there was greater female participation in the included studies, only two articles reported sex as an analysis variable in their data analysis [51, 53]. In addition, only one study addressed the result of this analysis [53], while none of the other studies explored differences in results when comparing sexes, even though these data were part of the characterization of participants in all studies, and considering that differences and physiological needs between sexes have already been reported in research [68, 69]. In this sense, future studies should prioritize analyzing results by gender to provide a more comprehensive understanding of how exergames influence mood. This approach would help identify potential differences in the effects experienced by men and women, offering valuable information about whether sex-specific factors play a role in the observed outcomes.

The participation of older women has been observed in other studies [70–73]. This phenomenon can be explained by the fact that women tend to take better care of their health compared to men, which may contribute to their greater representation in related studies.

Researchers [74] conducted a cross-sectional study based on 118,199 European individuals over 50 years of age and showed that women reported a slightly lower quality of life and more depressive symptoms than men. Furthermore, it was possible to conclude that the differences between the sexes increased with advancing age, regarding quality of life, while the differences in depressive symptoms between the sexes, remained stable between age groups. Thus, it should be noted that the differences between the sexes should be explored in future studies in order to cover a wider range of variables, such as understanding sex disparities in the context of quality of life and psychological factors, which could provide valuable insights for the development of new scientific research aimed at improving the general well-being of this population.

### Instruments used to assess the mood of the older adults

The selected studies used a variety of instruments to assess mood and its constructs, among them, only three used questionnaires that assess mood through various variables, which is the case of the Profile of Mood States (POMS) and BRUMS questionnaire [40, 52, 53].

The POMS instrument was developed by [75, 76] and has been shown to be reliable in assessing emotional states in different populations [77, 78]. The BRUMS is an adaptation of the POMS and was developed with the aim of allowing a rapid measurement of mood state [79]. The validations of this instrument for different populations attest to its quality [79, 80].

Considering the few studies that have investigated the mood profile, it is necessary for more articles to use gold standard instruments to assess mood, seeking to obtain more detailed results on the effects of exergames on the mood of older people, and subsequently, to evaluate the samples according to the new mood profiles [81, 82].

### Protocols and exergames used

The intervention protocols varied in time, session duration, relationship between control groups, consoles, and games applied. Therefore, it is crucial to discuss these differences and understand whether there is any predominant factor or one that should be further explored in future research.

The total duration of the sessions varied between 44 minutes on average [50] and 30 total hours of intervention [42], the session times varied between 30 [51, 54] and 60 minutes, and the number of sessions varied from 1 [50, 53] to 36 sessions [42]. In this way, the great diversity of protocols, together with the different mood assessments used, limit correlations between the results of the studies.

The most widely used consoles were the Xbox 360 with Kinect [40, 42, 45] and the Nintendo Wii [50, 52, 54], which are the two reference devices in the area of exergames and which are used in research in various areas, proving their efficiency [83, 84].

The most widely used exergame was the Wii Fit [50, 52, 54]. Other types of games that were recurrent among the studies were games that simulate sports, such as Wii Sports and Kinect Sports [42, 50]. These three exergames have a common characteristic, which is the variety of game styles that each one offers, which can vary between aerobic games, yoga, muscle exercises on the Wii Fit, and games inspired by tennis, baseball, bowling, golf, boxing, and athletics on Wii Sports and Kinect Sports, reducing the possibility that the player will lose interest in the game in the long term.

### Recommendations for the practice of exergames for older adults

Considering the nine included studies, experimental studies that verified the effects of practicing exergames on the mood states of the older adults and the summary of the evidence and knowledge developed from the analysis of the results, we can point out some recommendations and care recommendations for older adults. Thus, according to the authors’ recommendations, the practice of exergames is indicated for the older population and can serve as an alternative tool for the practice of physical exercises and rehabilitation of this population, and can be especially used in residential environments [51, 52, 54]. The main recommendations identified in the included studies are summarized in Frame 5.

**Frame 5:** Recommendations for the practice of exergames for the elderly.

**References**	**Recommendations**
[51]	It is recommended that the practice of moderate to vigorous intensity exergames be further explored as a tool to reduce health disparities experienced by the elderly population.
[40]	They did not present any recommendations.
[41]	The practice should be adapted to each player profile, and can be competitive, cooperative, more or less challenging; Researchers should ensure rest periods to help alleviate fatigue or pain in older adult participants.
[45]	Cognitive training simultaneously with exergames in the elderly is indicated.
[54]	It is recommended to use alternative physical activity tools in residential environments, such as nursing homes and care centers, for the practice of physical exercises and/or rehabilitation.
[53]	They did not present any recommendations.
[42]	They did not present any recommendations.
[52]	Training with exergames is recommended for the elderly population as it increases pleasure, promoting greater adherence and, consequently, effectiveness resulting from continuity.
[50]	Assess the appropriate level of in-game exertion and heart rate through appropriate sensor devices to ensure users are working at an intensity necessary for health benefit.

When playing, it is recommended that the practice be adapted to each player profile, which can be competitive or cooperative, and more or less challenging, thus favoring a more enjoyable environment for the player and promoting greater adherence and effectiveness resulting from the continuity of the practice [41, 52].

Considering the practical application of exergames in mental health disciplines, it is important to mention the accessibility and adaptability of these devices. They can be integrated into existing mental health interventions as a form of complementary therapy, particularly as an important complement to programs that target mood disorders, or as a preventive tool to maintain social well-being, reduce loneliness, and increase social connection in community or clinical settings [64], as the inclusion of the interactive and adaptive elements of the games can increase engagement and the effectiveness of interventions with this population [85, 86].

The implementation of exergames in clinical or community settings faces several barriers, such as the infrastructure needed to support exergames, including virtual reality equipment, which can be a challenge in terms of cost and maintenance, in addition to the lack of acceptance and familiarity of older adults with the technology, which can be overcome with adequate training and ongoing support. For this reason, the participation of health professionals and the adaptation of games to the specific needs of patients are crucial for the success of the implementation. [86–88].

It is also necessary for health professionals to take certain precautions in order to ensure the safety of practitioners. Some suggestions are that rest periods should be applied to help alleviate participants’ fatigue or pain [41]. In addition, simultaneous cognitive training with exergames in older adults is indicated [45]. Another precaution that should be considered is the assessment of the effort rate and heart rate during the practice of the game, which should ensure that users are working at an adequate and safe intensity, from moderate to vigorous, that provides better health benefits [50].

The authors’ recommendations for the production of new studies raise several relevant issues that should be emphasized. A recurring point is to conduct studies that include longer interventions, in order to be able to evaluate the long-term effects of the practice of exergames, and to be able to provide equipment so that older adults can practice exergames in their homes, reducing the barriers to practice and in the future enabling the generalization of the results found for the effects of interactive video games on healthy aging [41, 45, 50, 51, 54]. Obtaining a larger sample of older adults with greater racial and ethnic diversity and with a greater variety of depressive symptoms was also a recurring recommendation among studies [41, 42, 51]. A structured summary of these recommendations is presented in Frame 6.

**Frame 6:** Recommendations from the studies for future research.

**References**	**Recommendations**
[51]	Use methods to distribute interventional attention more evenly across groups (such as assigning twice-weekly check-in phone calls to the control group); Obtain a sample of older adults with more variation in depressive symptoms; Develop studies of the effectiveness of fully immersive virtual reality physical activity interventions among older adults; In future, larger trials that determine the effectiveness of the intervention in increasing physical activity, residents may be able to receive their virtual reality headset upon completion of the study.
[40]	They did not present any recommendations.
[41]	Explore how to generate more lasting effects from exergaming, such as increasing exergame accessibility at home; Consider recruiting more racially and ethnically diverse older participants.
[45]	Use accelerometers and heart rate monitors to quantify energy consumption and perceived subjective effort, and may also use self-report questionnaires to better interpret the results; Implement longer-lasting interventions; Explore the impact of this innovative technology, also comparing it with personalized training and conventional exercises, using a robust methodology to improve the quality of evidence and provide clear and reliable guidelines.
[54]	Studies with larger samples are needed to generalize the effects of interactive video games on healthy aging.
[53]	They did not present any recommendations.
[42]	Developing studies with a larger number of participants is necessary to demonstrate the perception of benefits from practicing exergames.
[52]	Develop studies that explore the relationship between training volume and control of body stability and balance
[50]	Develop research that explores the degree of variation in physical intensity (and therefore associated health benefits) of Wii Sport and Wii Fit games; Develop interventions that determine the effects of Wii use with repeated exposure on health and adherence; Develop longitudinal studies.

In addition to issues related to sample characteristics, the studies provided recommendations regarding data collection, such as the use of methods to disperse interventionist attention more equally between groups, such as assigning a routine of check-in telephone calls to the control group, which could be an effective solution for the intervention and control groups to receive more equal care [51]. Another suggestion is the use of equipment and methods that measure energy consumption and perceived subjective effort, in order to quantify the degree of variation in physical intensity [45, 50].

The recommendations consider the specificities of each study and their different objectives, and thus it is suggested that future studies address the effectiveness of fully immersive virtual reality physical activity interventions among older adults [51], compare personalized training and conventional exercises with exergames [45], explore the relationship between training volume and body stability and balance control [52], and determine the effects of Wii use on health and adherence [50].

## METHODS

This systematic review followed the recommendations of the Preferred Reporting Items for Systematic Reviews and Meta-Analyses literature search extension (PRISMA-S) [89, 90] and was registered in the Prospective Register of Systematic Reviews (PROSPERO), under number (CRD42024526448), before the completion of the selection of studies based on the eligibility criteria [91].

### Research strategy

The literature search was performed using the following electronic databases: PubMed (National Library of Medicine and National Institutes of Health), Embase, Web of Science, and Scopus. In the search for studies, the search terms were included in the “all fields” option of each database, except for Scopus, where the search filter “Article title, abstract, keywords” was used. The search terms used are described in Table 4.

**Table 4:** Characteristics of the studies and interventions performed in the included studies.

**References**	**Place of publication**	**Objective of the study**	**Study design**	**Groups**	**Protocol**	**Console**	**Exergame**
[51]	United States	To determine the feasibility of a virtual reality physical activity intervention among older adults, and to test the preliminary efficacy of the intervention in increasing physical activity and decreasing depressive symptoms.	Randomized pilot study	EG = exergame, goal setting and physical activity education CG = goal setting and physical activity education	Duration: 16 sessions of 40 minutes Frequency: 2x per week for 8 weeks	Recumbent bike Nordic Track R35^™^ and virtual reality headset Oculus Quest 2^™^	Holofit by Holidia^™^
[40]	Brazil	To evaluate the effects of a 12-week training program with Dance Exergames on the mood and functional fitness profile of elderly women.	Non-randomized experimental	EG = exergame only CG = manual activities workshops (cutting and sewing, painting and embroidery).	Duration: 24 sessions of 50 minutes Frequency: 2x per week for 12 weeks	XBOX 360 Kinect	Dance Central 3
[41]	United States	To understand older adults’ perceptions of the connections between an exergame intervention, “I Am Dolphin”, and their subjective well-being.	Non-randomized Qualitative Descriptive	FG = just exergame	Duration: 15 sessions of 60 minutes Frequency: 3x per week for 5 weeks	Bandit (motion detection sensor based on Kinect)	I Am Dolphin
[45]	Italy	To investigate the effects of cognitive-motor training with exergame on cognitive functions and mood in healthy elderly people.	Randomized controlled pilot study	EG = only exergame CG = kept daily routine	Duration: 8 sessions of 45 minutes Frequency: 3 to 4 times a week for 2 to 3 weeks	XBOX 360 Kinect	Fruit Ninja (habituation) Dr Kawashima: brain and body exercises
[54]	Turkey	To evaluate the effectiveness of interactive video games on mobility, general mood and quality of life and compare them with physical activity approaches in older adults.	Non-randomized controlled clinical trial	EG exergame = only exergame EG physical activity = training (cycling on a stationary bike and walking on a treadmill) CG = maintained daily routine	Duration: 16 sessions of 30 minutes Frequency: 2x per week for 8 weeks	Nintendo Wii	“Wii Fit Plus” (Wii Fit^™^ and Wii Balance Board^™^, Nintendo of Europe GmBH Grossostheim, Deutschland)
[53]	Brazil	To compare the performance of physically active and sedentary elderly people in exergames.	Non-randomized cross-sectional	AG = just exergame SG = just exergame	Duration: 1 session (time varies depending on performance)	Computer or Notebook with webcam	MoviLetrando
[42]	Brazil	To investigate the perception of people aged 55 or over regarding the benefits of an exercise program with exergames.	Qualitative Randomized exploratory	FG = just exergame performed in pairs	Duration: 36 sessions of 50 minutes Frequency: 3x per week for 12 weeks	Xbox 360 Kinect	Kinect Sports Ultimate Collection (athletics, bowling, boxing, skiing, football, tennis, table tennis and some mini games)
[52]	Brazil	To verify the effect of exercises with Nintendo Wii on chronic low back pain, functional capacity and mood of elderly women.	Double-blind randomized controlled trial	CG physical activity = strength exercises and core training EG exergame = strength exercises and core training and exergame	Duration: 24 sessions, general minutes NF, the exergame lasted 30 minutes. Frequency: 3x per week for 8 weeks	Nintendo Wii	“Wii Fit Plus” (Wii Fit^™^ and Wii Balance Board^™^)
[50]	England	Reports the results of an exploratory study that examined the suitability and potential benefit of the Nintendo Wii as a tool for promoting physical activity for older adults.	Non-randomized exploratory	EG = exergame	Duration: 1 session (time varies, average 44±5.0 minutes)	Nintendo Wii	Wii Sports and Wii Fit (aerobic step, bowling, hula hooping, running, torso and waist twisting, squat with rowing, leg extension, yoga breathing and half-moon yoga pose)

Abbreviations: n: number; S: samples; EG: experimental group; CG: control group; AG: active group; SG: sedentary group; FC: Focus Group; M: masculine; F: female.

### Manual search

In addition, the authors searched the reference lists of all identified studies to find any further relevant articles [92].

### Eligibility criteria

The search was carried out in August 2024. To obtain a greater number of publications, there was no time limit for searching for published articles. The eligibility and exclusion criteria are described in Table 5 and were based on the PICOS strategy: Population, Intervention, Comparator, Outcome, and Study Design [48].

**Table 5:** Quality assessment for experimental study.

**ID Studies**	Criteria^*^												**Total score**	**Quality rating**
	1	2	3	4	5	6	7	8	9	10	11	12		
51	1	1	1	1	1	1	1	0	1	1	0	0	9	Regular
40	1	1	1	1	0	1	1	0	0	1	0	1	8	Regular
41	1	1	1	1	0	1	1	0	1	1	0	0	8	Regular
45	1	1	1	1	1	1	1	0	1	0	0	0	8	Regular
54	1	1	1	1	1	1	1	0	0	1	0	0	8	Regular
53	1	1	1	1	0	1	1	0	1	1	0	0	8	Regular
42	1	1	1	1	0	1	1	0	0	0	0	0	6	Regular
52	1	1	1	1	1	1	1	1	1	1	0	0	10	Regular
50	1	1	1	1	0	1	1	0	1	1	0	0	8	Regular
Agreement % (Kappa)	100	100	100	100	75	100	100	100	75	100	100	100	95.83
Kappa interpretation (agreement)	Almost perfect	Almost perfect	Almost perfect	Almost perfect	Moderate	Almost perfect	Almost perfect	Almost perfect	Moderate	Almost perfect	Almost perfect	Almost perfect	Strong

No or cannot determine or not applicable or not reported (0); Yes (1); The quality of the studies was classified as poor (0–4 out of 12 questions), Regular (5–10 out of 12 questions), or good (11–12 out of 12 questions). ^*^Criteria: (1) Was the study question or objective clearly stated? (2) Were eligibility/selection criteria for the study population prespecified and clearly described? (3) Were the participants in the study representative of those who would be eligible for the test/service/intervention in the general or clinical population of interest? (4) Were all eligible participants that met the prespecified entry criteria enrolled? (5) Was the sample size sufficiently large to provide confidence in the findings? (6) Was the test/service/intervention clearly described and delivered consistently across the study population? (7) Were the outcome measures prespecified, clearly defined, valid, reliable, and assessed consistently across all study participants? (8) Were the people assessing the outcomes blinded to the participants' exposures/interventions? (9) Was the loss to follow-up after baseline 20% or less? Were those lost to follow-up accounted for in the analysis? (10) Did the statistical methods examine changes in outcome measures from before to after the intervention? Were statistical tests done that provided p values for the pre-to-post changes? (11) Were outcome measures of interest taken multiple times before the intervention and multiple times after the intervention (i.e., did they use an interrupted time-series design)? (12) If the intervention was conducted at a group level (e.g., a whole hospital, a community, etc.) did the statistical analysis take into account the use of individual-level data to determine effects at the group level?

Studies that met all of the following criteria were included in the review: (a) Population: Older adults; (b) Study design: Experimental, randomized, non-randomized, pre- and post-treatment studies; (c) Subjects: Older individuals aged 60 years or older; (d) Outcomes: Effects of exergames on mood state; (e) Country/area: Any country and region; (e) Type of article: Peer-reviewed publications; (f) Language: Restricted to publications of articles written in English, Portuguese, and Spanish.

The following studies were included: Experimental, randomized, non-randomized, pre and post studies. The following studies were excluded from the review: Reviews, letters, editorials, comments, abstracts, conference proceedings, study protocols, and articles not available in full after request by email contact with the authors.

The exclusion of non-experimental, cross-sectional, and longitudinal studies strengthens the internal validity of the current study by minimizing bias and confounding factors. Cross-sectional studies capture only short-term effects within a single session, limiting causal inference, while longitudinal designs may introduce variability due to differing follow-up periods and external factors [93]. Our study sought to prioritize experimental designs, including randomized and non-randomized trials, with the intention of ensuring a controlled evaluation of the effects of exergames on mood, reducing heterogeneity and enhancing the comparability of results [61, 71].

### Study selection

After the searches, two independent researchers (CBG and WMC) selected potentially relevant studies based on the titles. The selected studies went through the phases of reading the abstract, followed by reading in full, when the inclusion/exclusion criteria were applied. If there was a divergence of opinion among the researchers in any of the phases, this was decided through discussion among them, and if no agreement was reached, the third researcher (AA) made the final decision in a consensus meeting. The selection was performed using Rayyan CRQI [94]. The references of the included articles were reviewed to identify potentially relevant articles.

### Data extraction

Data extraction and synthesis were performed by analyzing the characteristics of the participants (sample, sex and age group), the instrument used to assess mood and data extraction and synthesis were performed by analyzing the characteristics of the participants (sample, sex, and age group), the instrument used to assess mood, and the experimental protocol (intervention time, weekly frequency, number of sessions, console, and exergame). Regarding mood assessment, the results of the studies were organized into categories: positive effects, no effects, and negative effects.

### Evaluation of study quality

The quality of the included studies was assessed using the Quality Assessment Tool for Before-After (Pre-Post) from the National Institutes of Health – [55]. In the present study, this tool was used for the methodological evaluation of the studies. At the end of the evaluation, the instrument presents a final, based on each criterion answered; for a “yes” answer, one point is added, and for “no”, “not applicable”, “not reported” or “cannot determine” answers, the value assigned is zero. The experimental studies tool (pre-post) contains 12 criteria, thus the overall score ranges from 0–12. A higher score indicates better study quality [55].

The assessment was performed independently by two reviewers (CBG and WMC). Disagreements were resolved by consensus. When necessary, a third author (AA) was asked to decide the final opinion. The quality of the studies was not considered as an inclusion or exclusion criterion. Previous studies have used these tools, demonstrating satisfactory applicability [95, 96]. In addition, the degree of agreement between the two evaluators was analyzed using Cohen’s Kappa test. For interpretation, the agreement classification approach was used, as follows: None 0–4%; minimum 4–15%; weak 15–35%; moderate 35–63%; strong 64–81%; and almost perfect 82–100% [97].

## Limitations and Future Studies

Despite the significant findings, our evidence is limited by the variations in mood assessment instruments used, heterogeneity across intervention durations and types, limited information regarding the participants’ characteristics, and small number of studies. These factors made it impossible to determine whether any specific exergame is more effective in improving the mood of older adults and whether there are differences in the effects of different durations and frequencies of exergame practice in this population. To obtain more reliable results, future studies should focus their database searches on a single type of exergame or console, in order to analyze the specific effects of these instruments on mood and evaluate whether different games or consoles influence study outcomes.

Additionally, we encourage the use of validated and standardized tools to assess mood in future research, as this would enhance the robustness and consistency of result comparisons between studies. Future research could also consider mental health factors, sex, race, and ethnicity to explore whether these variables influence the effects of exergames on mood. Moreover, studies should aim to identify which types of exergames are most beneficial for different individuals, and include interventions with longer durations and/or higher frequencies, to address this gap in the literature and provide insights into the long-term effects of exergames on the mood of older adults. To strengthen the representativeness and external validity of the findings, it is crucial to include larger and more diverse samples, as well as to consider additional strategies to assess and mitigate publication bias. These considerations have been incorporated into our discussion of limitations to appropriately address these observations. By adopting these approaches, future studies will contribute to a more accurate and comprehensive understanding of the impacts of exergames, including personalized and effective interventions for diverse populations.

## Innovations, Strengths, and Applications

The current study presents innovative aspects because it is the first systematic review to address the effects of exergames on the mood of older individuals, analyzing only experimental intervention studies, and thus allowing for more specific and controlled results to be observed. In addition, it was possible to analyze different variables that make up the mood related to the mental health of older individuals. Thus, the results reported offer an opportunity for physical education professionals or physiotherapists who work directly with older individuals to discuss and integrate exergames into their practice, possibly bringing greater motivation and interest in physical exercise for these individuals.

## CONCLUSION

The results indicate that the practice of exergames had a positive effect on the mood of older adults, reducing depressive symptoms, anger, confusion, tension, and fatigue, and promoting engagement and immersion during practice and socialization. Among the reviewed studies, no negative effects on the mood states of the older adults were reported after participating in experiments with exergames, suggesting that this activity can be recommended for this group without known risks. However, given the number of experiments carried out to date, it is recommended that new studies involving this population be carried out, with long-term designs, aiming to increase the level of available evidence.
